# NM23-H1 expression of head and neck squamous cell carcinoma in association with the response to cisplatin treatment

**DOI:** 10.18632/oncotarget.1912

**Published:** 2014-04-18

**Authors:** Yi-Fen Wang, Chun-Ju Chang, Jen-Hwey Chiu, Chin-Ping Lin, Wing-Yin Li, Shyue-Yih Chang, Pen-Yuan Chu, Shyh-Kuan Tai, Yu-Jen Chen

**Affiliations:** ^1^ Department of Otorhinolaryngology and Head and Neck Surgery, Taipei Veterans General Hospital, Taipei, Taiwan; ^2^ Department of Medicine, National Yang Ming University, Taipei, Taiwan; ^3^ Department of Food Science, National Taiwan Ocean University, Keelung, Taiwan; ^4^ Institute of Traditional Medicine, National Yang Ming University, Taipei, Taiwan; ^5^ Department of Medical Research, Mackay Memorial Hospital, Taipei, Taiwan; ^6^ Department of Pathology, Taipei Veterans General Hospital, Taipei, Taiwan; ^7^ Department of Radiation Oncology, Mackay Memorial Hospital, Taipei, Taiwan

**Keywords:** Head and neck squamous cell carcinoma (HNSCC), NM23-H1, Metastasis, Cisplatin, Prognosis

## Abstract

We recently reported that low NM23-H1 expression of head and neck squamous cell carcinoma (HNSCC) correlated with poor patients' prognosis. Growing evidence has indicated that high tumor NM23-H1 expression contributes to a good response to chemotherapy. Therefore, we investigated the role of NM23-H1 in susceptibility of HNSCC cells to cisplatin and its clinical significance, as well as the *in vitro* study for validation was performed. Using immunohistochemistry, we analyzed NM23-H1 expression in surgical specimens from 46 HNSCC patients with cervical metastases receiving surgery and adjuvant chemoradiotherapy. Low tumor NM23-H1 expression correlated with locoregional recurrence of HNSCC following postoperative cisplatin-basedtherapy (p = 0.056) and poor patient prognosis (p = 0.001). To validate the clinical observation and the effect of NM23-H1 on cisplatin cytotoxicity, we established several stable clones derived from a human HNSCC cell line (SAS) by knockdown and overexpression. Knockdown of NM23-H1 attenuated the chemosensitivity of SAS cells to cisplatin, which was associated with reduced cisplatin-induced S-phase accumulation and downregulation of cyclin E1 and A. Overexpression of NM23-H1 reversed these results, indicating the essential role of NM23-H1 in treatment response to cisplatin. NM23-H1 may participate in HNSCC cell responses to cisplatin and be considered a potential therapeutic target.

## INTRODUCTION

Head and neck squamous cell carcinoma (HNSCC) is the most common malignant pathology, with an incidence ranging from <0.1% to >40% worldwide [1, 2]. In Taiwan, HNSCC has been the fourth or fifth leading cause of cancer death in the men the seventh among the entire population since 1991 [3].

Despite the use of multiple therapeutic modalities, the overall survival of HNSCC patients has not improved in the last two decades [4]. One major cause of treatment failure is early metastases and poor response of HNSCC cells to chemoradiation. Clinical surveys showed that more than half of HNSCC patients with resectable tumors had lymphatic metastases at diagnosis [5, 6]. Therefore, it is relevant to assess whether patients with metastases respond well to cisplatin-based chemoradiation. Biomarkers for predicting the response to anticancer agents are imperative to initiate optimal treatments and improve therapeutic outcomes.

The *nm23* gene was identified by differentiating cDNA libraries from murine melanoma-derived cell lines with different metastatic potentials. High expression of NM23 was found in weakly metastatic cancer cell lines [7]. The human *nm23* (*nme*) family consists of at least ten members and low NM23-H1 expression has been shown to correlate with metastasis and chemoresistance of some cancers [8-11]. However, clinical studies on NM23-H1 expression in different cancers did not draw a consistent conclusion [11-16]. This discordance might arise from the inconsistency among tumor types, specificity of antibodies and scoring system used for immunohistochemical interpretation. It is worth noting that carcinogenesis is a multifactorial process: a specific factor might play a certain role within a defined period of disease progression, and its effects might be different in other periods. In our earlier observation, there was a dynamic change that increased NM23-H1 protein in the primary tumors followed by a decreased level in the metastatic lymph nodes [6, 17]. Metastatic cells with low NM23-H1 expression should arise from an NM23-H1-deficient and genetically unstable subpopulation within the primary cancer [18]. NM23-H1 expression might be gradually reduced over the course of tumor development [19]. For example, an initial decrease in NM23-H1 levels can promote cancer metastases and further loss may confer chemoresistance. NM23-H1exhibits 3′-5′ exonuclease activity potentially involved in DNA repair and its expression is low in metastatic cells, inferring that such reduced protein level may allow these cells to escape from apoptosis [19-22]. This survival mechanism of metastatic cells may also contribute to chemoresistance of recurrent tumors and poor patient prognosis [8]. Therefore, potential therapeutic interventions might include restoring NM23-H1 expression to surmount the resistance of cancer cells to anticancer agents [23].

There is a paucity of studies concerning the prognostic significance of NM23-H1 for cancer patients with resectable metastases treated by postoperative chemoradiation. We recently noted that low NM23-H1 expression of HNSCCs correlated with the occurrence of lymphatic metastases and its protein level indeed was reduced in the metastatic tumors [17]. To clarify the involvement of NM23-H1 in response of residual minimal disease to adjuvant therapy, we assessed the correlation between NM23-H1 expression and clinicopathologic parameters, specifically focusing on HNSCC patients with resectable metastases treated by cisplatin-based chemoradiotherapy after therapeutic surgery. To validate the effect of NM23-H1 on cisplatin cytotoxicity in HNSCC cells, we established stable clones derived from a human HNSCC SAS cell line by both knockdown and overexpression of NM23-H1. Following the analyses, we deduced the role of NM23-H1 in chemosensitivity of HNSCC cells to cisplatin.

## RESULTS

### Low NM23-H1 expression in HNSCC tumors correlated with poor prognosis of patients treated with postoperative chemoradiation

To clarify the role of NM23-H1 in the prognosis of HNSCC patients with resectable metastases treated by postoperative cisplatin, we examined NM23-H1 expression in the surgical tumor specimens. On immunochemistry, NM23-H1 localized predominantly in the cytoplasm and focally in the nucleus; however, we focused primarily on its nuclear expression because of the findings of recent studies [24, 25]. Our analysis included 46 HNSCC patients with cervical metastasis treated by surgery and postoperative cisplatin-based chemoradiation. The clinical relevance of tumor NM23-H1 expression was assessed in comparison with clinicopathologic features, including age, primary tumor size, nodal involvement, distant metastasis and tumor recurrence (Table 1). Seventy-four percent (14/19) of patients with locoregional recurrence had NM23-H1-negative tumors compared with 41% (11/27) of patients without recurrence. Chi-square test with Yates( correction showed that more patients with NM23-H1-negative tumors (14/25) had tumor recurrence than those with NM23-H1-positive tumors (5/21), with marginal significance (p=0.056).

**Table 1:** Relationship between NM23-H1 expression in head and neck squamous cell carcinoma and clinicopathologic parameters of 46 patients with resectable cervical metastasis treated by postoperative cisplatin-based chemoradiation

Clinicopathologic parameters	Number of patients	Interpretation of tumor NM23-H1 expression		
		Negative	Positive	p value^1^
Age (years)				
≤50	26	15	11	0.825
>50	20	10	10	
Primary tumor size				
≤4 cm	29	18	11	0.286
>4 cm	17	7	10	
Metastatic lymph node(s)				
=1	5	2	3	0.836
>1	41	23	18	
Distant metastasis				
Negative	34	16	18	0.182
Positive	12	9	3	
Tumor recurrence				
Negative	27	11	16	0.056
Positive	19	14	5	

1 Based on Chi-square test with Yates' (continuity) correction

**Figure 1:**
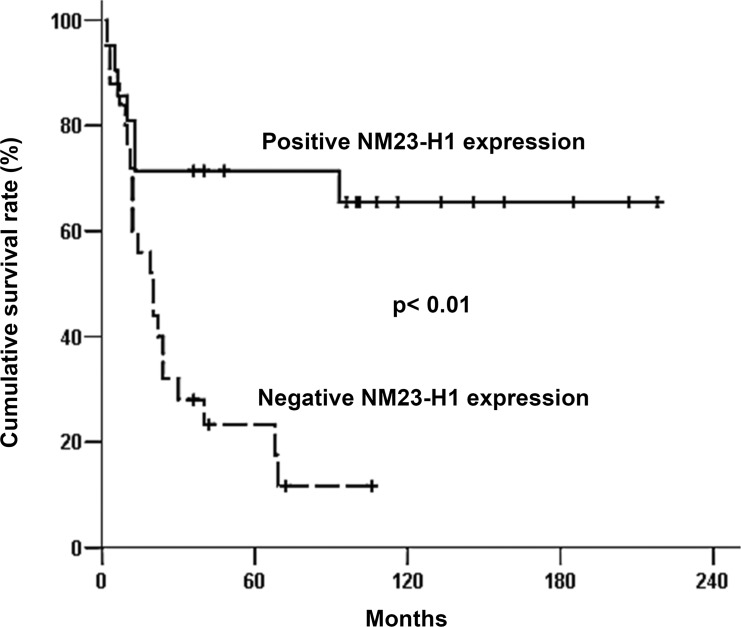
Overall survival curve of 46 patients with head and neck squamous cell carcinoma associated with NM23-H1 expression of primary tumors Survival analysis was performed using the Kaplan–Meier method. By log-rank test, we assessed the difference in survival time between patients with immunohistochemically NM23-H1-positive and NM23-H1-negative tumors. Patients with NM23-H1-positive tumors had significantly longer survival time than those with NM23-H1-negative tumors (p<0.01).

To determine whether a low NM23-H1 level affects on clinical outcome, we evaluated the prognostic significance of tumor NM23-H1 expression in HNSCC patients using the Kaplan−Meier method. On univariate analyses using log-rank tests, patients with larger primary tumors (p=0.030), locoregional recurrence (p<0.01), and distant metastasis (p<0.01) demonstrated poorer survival. Based on immunohistochemistry, patients with NM23-H1-negative tumors displayed a less favorable outcome than those with NM23-H1-positive tumors (p<0.01; Figure 1). On multivariate analyses using a Cox proportional hazard model, distant metastasis (p<0.01) remained an independent factor associated with patient prognosis. However, tumor NM23-H1 expression was marginally associated with patient prognosis, and did not reach statistical difference (p=0.083; Table 2).

**Table 2:** Survival analysis of 46 patients with head and neck squamous cell carcinoma with resectable cervical metastasis treated by postoperative cisplatin-based chemoradiation

Clinicopathologic parameters (Number of patients analyzed)	p value	
	Univariate analysis^1^	Multivariate analysis^2^
Age (years)	0.781	
≤50 (26)		
>50 (20)		
Primary tumor size		
≤4 cm (29)	0.030	0.263
>4 cm (17)		
Metastatic lymph node(s)		
=1 (5)	0.851	
>1 (41)		
Distant metastasis		
Negative (34)	< 0.001	0.008
Positive (12)		
Tumor recurrence		
Negative (27)		
Positive (19)	0.003	0.136
NM23-H1 expression in primary tumor		
Negative (25)		
Positive (21)	0.001	0.083

1 Based on Log-rank test

2 Based on Cox proportional hazards model

### Decreased NM23-H1 expression level by knockdown and increased NM23-H1 expression level by overexpression

**Figure 2:**
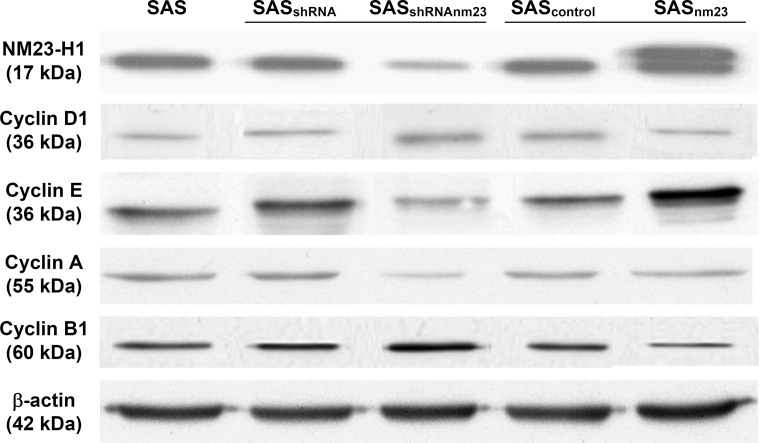
Western blot analysis of the protein levels of NM23-H1 and cyclin D1, E, A1, and B1 in the SAS head and neck squamous cell carcinoma clones Knockdown of NM23-H1 downregulated cyclin E and A, and slightly upregulated cyclin D1 and B1 in SAS_shRNAnm23_ cells, compared with SAS_shRNA_. β-actin served as a loading control. Abbreviations: Parent SAS clone, SAS; mock knockdown clone, SAS_shRNA_; NM23-H1 knockdown clone, SAS_shRNAnm23_; mock overexpression clone, SAS_control_; NM23-H1 overexpression clone, SAS_nm23_.

In the earlier study, we established several stable clones derived from a human HNSCC SAS cell line by knockdown and overexpression [17]. Knockdown was achieved by transfection of a pSuper plasmid carrying the shRNA sequence targeted to *nm23-H1* and pSuper alone as a control into the SAS cell line. After selection, SAS_shRNAnm23_ (carrying *nm23-H1* shRNA) and SAS_shRNA_ (carrying the pSuper plasmid) clones were obtained. In addition, SAS clones stably expressing the ectopically introduced HA-tagged NM23-H1 and harboring a control plasmid were also established, designated as SAS_nm23_ and SAS_control_. NM23-H1 expression in these cell clones was examined by Western blot (Figure 2). The NM23-H1 protein level of SAS_shRNA_ and SAS_control_ remained similar to that of parental SAS cells whereas that of SAS_shRNAnm23_ was decreased by 75% compared with the mock SAS_shRNA_. Overexpression of the ectopically introduced HA-tagged NM23-H1 was detected as a slightly upshifted molecular weight signal.

### Knockdown of NM23-H1 downregulated cyclins E and A

To address the potential physiologic relevance of NM23-H1 proteins in SAS cells, we evaluated whether NM23-H1 could modulate the expression of cyclin D1, E, A and B1. On western blot, knockdown of NM23-H1 downregulated cyclin E and A, whereas overexpression of NM23-H1 upregulated them, compared with the mock controls. In addition, knockdown of NM23-H1 slightly increased the protein levels of cyclin D1 and B1, while overexpression of NM23-H1 marginally increased them. These results suggest that NM23-H1 plays a role in modulating cyclin expression (Figure 2).

### Knockdown and overexpression of NM23-H1 did not affect cellular proliferation and cell cycle distribution

**Figure 3:**
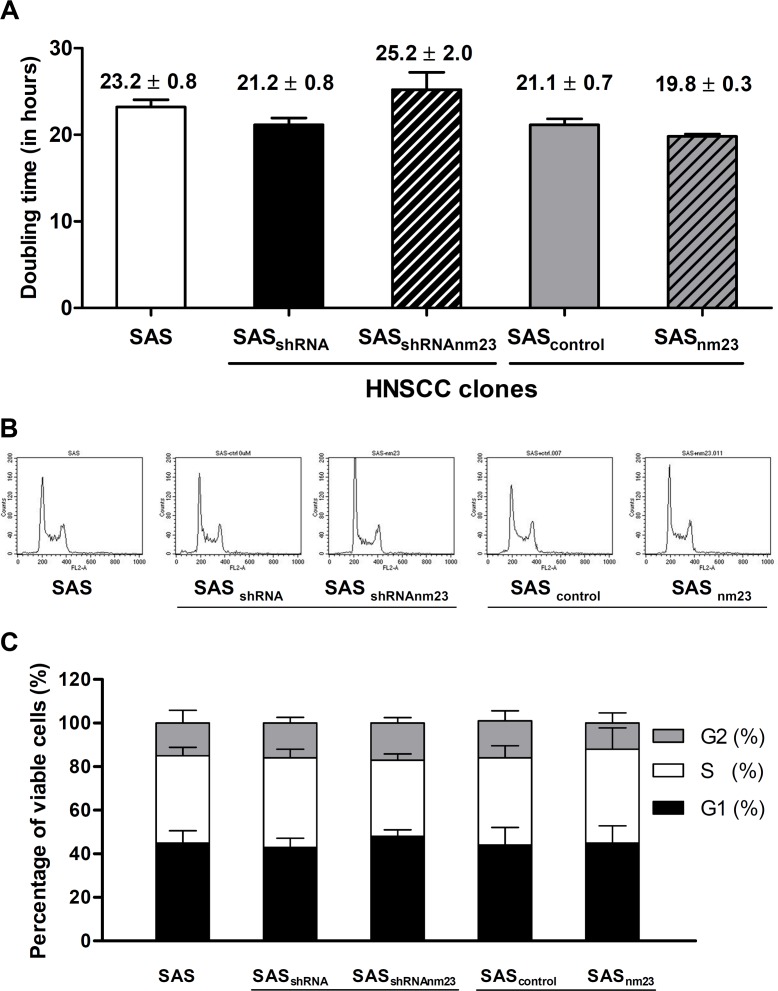
Knockdown and overexpression of NM23-H1 did not affect cellular proliferation of SAS cells A, doubling time. Cell numbers were assessed by trypan blue exclusion assay and doubling time was determined by calculating growth rates during exponential growth. B, cell cycle analysis. SAS cells were grown, synchronized with thymidine, released in fresh medium for 24 hours, and then subjected to cell cycle analysis to determine their DNA content. C, cell cycle distribution. Percentage of cells in each phase of the cell cycle was determined by deconvolution of the DNA content-frequency histogram. The data shown represent the mean ± standard error of three independent experiments.

To define the effect of NM23-H1 expression on the growth kinetics of SAS cells, we evaluated proliferation rates by trypan blue exclusion assays. There was no significant difference in doubling time among the SAS clones with various levels of NM23-H1 expression, revealing that NM23-H1 expression did not affect their proliferative capacity (Figure 3A).

To explore the possibility of a subtle effect on cellular proliferation following knockdown or overexpression of NM23-H1, cell cycle analysis was performed using flow cytometry. As shown in Figure 3B, normal cell cycle progression was observed in all SAS clones. Among these clones, there was no significant difference in cellular distribution of G0-G1, S and G2-M phases (Figure 3C).

### Knockdown of NM23-H1 attenuated the susceptibility of SAS cells to cisplatin

**Figure 4:**
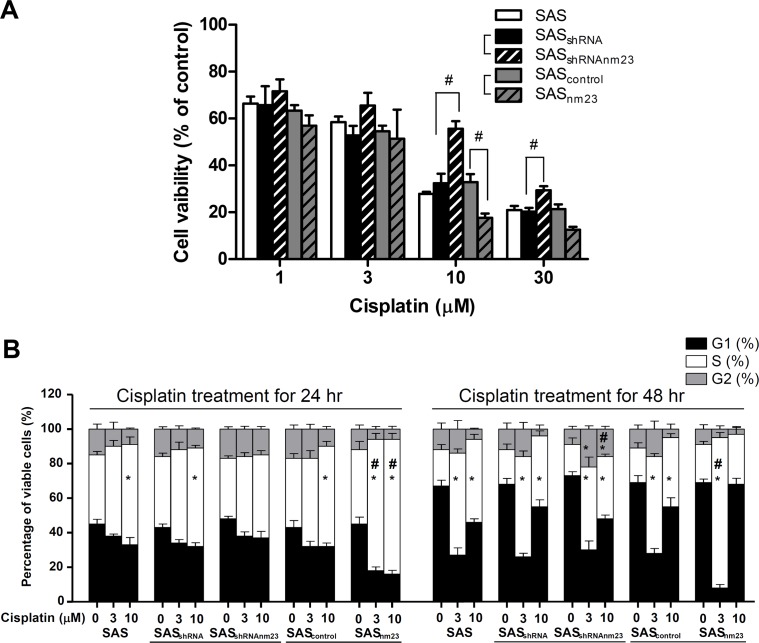
Knockdown of NM23-H1 attenuated the susceptibility of SAS cells to cisplatin and delayed cisplatin-induced S-phase cell accumulation A, cell viability. SAS cells were treated with 1, 3, 10, or 30 μM cisplatin for 48 hours. Cell viability was determined by trypan blue dye exclusion assay. B, cell cycle analysis. SAS cells were treated with cisplatin for 24 or 48 hours. The cell populations in each cell cycle phase were analyzed by flow cytometry. Data represent the mean ± standard error of three independent experiments. *p<0.05 compared with the vehicle control; #p< 0.05 compared with the mock control; statistical significance was determined using two-way analysis of variance.

To elucidate the role of NM23-H1 in SAS cell chemosensitivity, cell viability was assessed using trypan blue exclusion assays following 48-hour treatment with increasing concentrations of cisplatin (0, 1, 3, 10, and 30 μM). The viability of NM23-H1-knockdown (SAS_shRNAnm23_) cells was significantly higher than that of the mock control (SAS_shRNA_) upon exposure to cisplatin at 10 and 30 μM, indicating that knockdown of NM23-H1 attenuates its cytotoxicity. Conversely, the viability of NM23-overexpressing (SAS_nm23_) cells was significantly lower than that of the mock control (SAS_control_) when they were treated with cisplatin at 10 μM (Figure 4A). The 50% cell growth inhibition concentration (IC50) of cisplatin was 14.50 0.37 M for SAS_shRNAnm23_, significantly higher than 4.07 ± 0.22 μM for SAS_shRNA_ cells (p<0.01). In contrast, the IC50 of cisplatin for SAS_nm23_ was 2.63 ± 0.31 μM, lower than 5.62 ± 0.29 μM for SAS_control_ cells (p*<*0.01).

### Early apoptotic DNA fragmentation was not detected after cisplatin treatment

To ascertain whether cisplatin treatment leads to early apoptosis of SAS cells, we conducted a DNA fragmentation assay. After exposure of SAS cells to cisplatin at 0, 3, 10, and 30 μM for 24 and 48 hours, there was no DNA ladder observed, in contrast to the positive control treated with cycloheximide (data not shown). This result indicated that classical early apoptosis might be not the major pathway of cisplatin-induced cell death in SAS cells.

### SAS cells exposed to cisplatin primarily accumulated in S phase

We treated SAS cells with various concentrations of cisplatin (0, 3, and 10 μM) for increasing periods (24 and 48 hours) and analyzed the cell cycle by flow cytometry. Cell cycle arrest in a given phase of the cell cycle is defined as when cell numbers in that phase increase above the levels of vehicle controls [26]. Based on this limited criterion, the exposure of SAS cells to cisplatin primarily induced S-phase arrest with a decrease in G1 proportion. Upon 48-hour cisplatin treatment at the higher concentration of 10 μM, there was an increase in the G1-phase population of SAS cells compared with that at 3 μM (Figure 4B).

### Knockdown of NM23-H1 delayed cisplatin-induced S-phase accumulation

Compared with vehicle controls, 24-hour exposure to 10-μM cisplatin caused a significant increase in the S-phase fraction (SPF) of most SAS clones except NM23-H1-knockdown (SAS_shRNAnm23_) cells. Knockdown of NM23-H1 delayed cisplatin-induced S-phase arrest until the later time point of 48 hours and allowed more SAS_shRNAnm23_ cells to progress to G2 phase compared with the mock control (SAS_shRNA_). On the other hand, overexpression of NM23-H1 augmented the S-phase accumulation of SAS_nm23_ cells compared with the mock control (SAS_control_) with cisplatin treatment at 3 μM for 24 and 48 hours, as well as at 10 μM for 24 hours (Figure 4B).

### NM23-H1 expression was upregulated following cisplatin treatment

**Figure 5:**
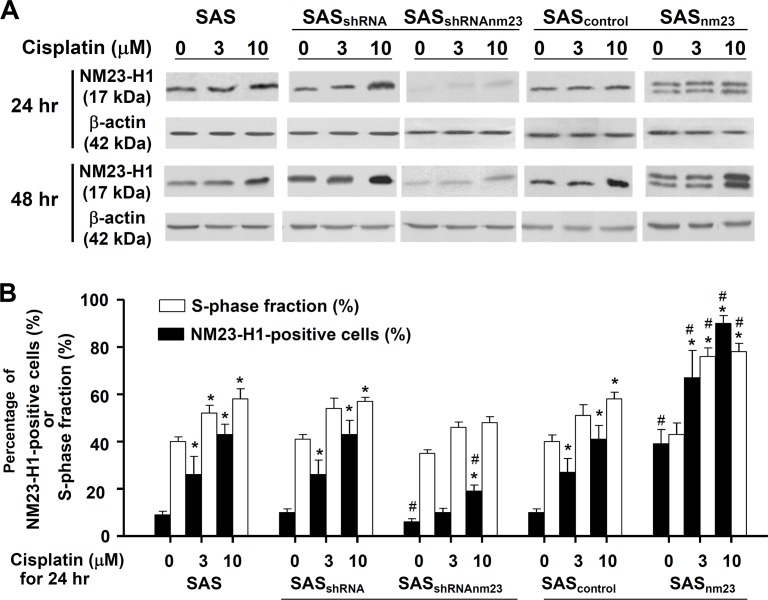
NM23-H1 expression of SAS cells was upregulated upon cisplatin treatment A, western blot analysis. Cells were treated with cisplatin at 3 or 10 μM for 24 or 48 hours. Western blot analysis of the protein levels of NM23-H1 in the SAS clones. B, cell cycle analysis. Following 24-hour exposure to cisplatin at 3 or 10 μM, cells were fixed and stained with an anti-NM23-H1 antibody and propidium iodide. Percentage of NM23-H1-positive cells and S-phase fraction in the SAS clones were determined by flow cytometry. The bar graph demonstrates the positive correlation between the percentage of NM23-H1-positive cells and the S-phase fraction in the SAS clones. Each bar represents the mean ± standard error of three independent experiments. *p<0.05 compared with the vehicle control; #p<0.05 compared with the mock control; statistical significance was determined using two-way analysis of variance.

To establish the relationship between NM23-H1 expression and cisplatin-induced cytotoxicity, we examined the NM23-H1 levels of SAS cells by Western blot. Following cisplatin treatment, there was a time- and concentration-dependent increase in NM23-H1 expression, with the response of NM23-H1-overexpressing (SAS_nm23_) cells being the most pronounced (Figure 5A). The levels of cisplatin-induced NM23-H1 accumulation in SAS cells were further confirmed quantitatively using flow cytometry. Following 24-hour exposure to cisplatin, the population of NM23-H1-positive cells was significantly larger than that of vehicle controls in the SAS clones with the exception of SAS_shRNAnm23_ after 3-μM cisplatin treatment. In addition, a significant increase in the S-phase fraction followed NM23-H1 accumulation in SAS cells, suggestive of an association between NM23-H1 accumulation and S-phase arrest after cisplatin treatment (Figure 5B).

### Knockdown of NM23-H1 reduced cyclin E accumulation and maintained a low cyclin A level upon cisplatin treatment

**Figure 6:**
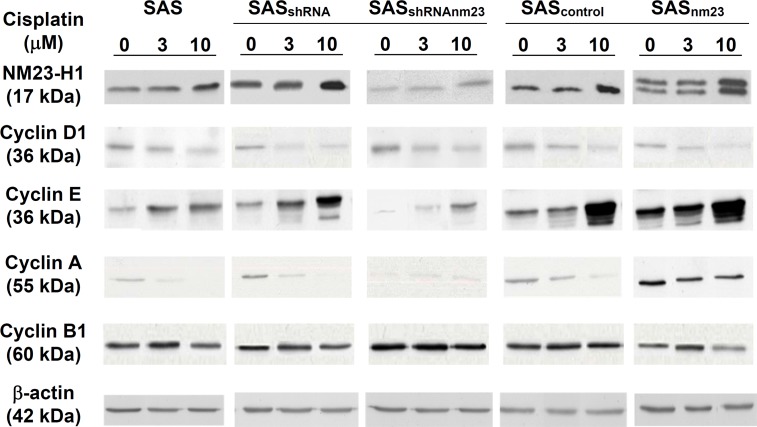
Western blot analysis showing the effect of NM23-H1 expression on the protein levels of cyclin D1, E, A1, and B1 upon cisplatin treatment Following 48-hour exposure to cisplatin at 3 or 10 μM, cells were collected, lysed, and analyzed by western blot for the SAS clones, including parental (SAS), mock knockdown (SAS_shRNA_), NM23-H1 knockdown (SAS_shRNAnm23_), mock overexpression (SAS_control_), and NM23-H1 overexpression (SAS_nm23_) cells. Knockdown of NM23-H1 reduced cisplatin-induced accumulation of cyclin E, as well as maintained cyclin A at a relatively low level in SAS_shRNAnm23_ cells, compared with the SAS_shRNA_. β-actin served as a loading control.

Following 48-hour cisplatin exposure, SAS cells displayed decreased cyclin D1 and increased cyclin E levels in a concentration-dependent manner compared with the vehicle controls (Figure 6). After cisplatin treatment, NM23-H1-overexpressing (SAS_nm23_) cells exhibited a greater increase in cyclin E levels, whereas NM23-H1-knockdown (SAS_shRNAnm23_) cells showed a less pronounced increase compared with their mock controls. Upon cisplatin exposure, cyclin A expression was concentration-dependently reduced in most SAS clones, with the exception of SAS_shRNAnm23_, which maintained a relatively low expression level. Cyclin B1 was slightly increased upon cisplatin treatment at 1 μM and decreased at 10 μM compared with the vehicle controls. At the higher concentration of 10 μM cisplatin, SAS_nm23_ cells presented a larger decrease in cyclin B1 expression than the mock control, while SAS_shRNAnm23_ maintained it at a relatively high level.

## DISCUSSION

In patients with cervical metastases receiving postoperative cisplatin-based chemoradiation, low NM23-H1 expression in HNSCC tumors correlated with locoregional recurrence, further indicative of poor prognosis. Following knockdown and overexpression of NM23-H1 in the human HNSCC SAS cell line, cells with low NM23-H1 expression were less susceptible to cisplatin than those with high NM23-H1 expression. The cisplatin-induced death of SAS cells resulted from prolonged S-phase arrest, possibly associated with NM23-H1 accumulation in response to DNA damage. It is likely that knockdown of NM23-H1 downregulated cyclin E and A in SAS cells, subsequently leading to less S-phase arrest and cell death following cisplatin treatment.

Analysis of clinical information showed that low tumor NM23-H1 expression was a poor survival indicator and correlated with locoregional recurrence of HNSCC in patients with metastases treated with cisplatin postoperatively. This finding is concordant with an earlier report on patients with esophageal squamous cell carcinoma undergoing cisplatin-based chemotherapy following resection [27]. To our knowledge, there are few articles reported about the role of NM23-H1 in response to postoperative chemoradiation in HNSCC patients with metastases [17]. We observed a correlation between low NM23-H1 expression and locoregional recurrence of HNSCC tumors, conceivably as a consequence of poor response to cisplatin-based therapy.

The present laboratory experiments showed that suppression of NM23-H1 expression attenuated cisplatin cytotoxicity, and elevation of NM23-H1 expressionenhanced the chemosensitivity of SAS cells. Both the clinical observation and *in vitro* study results supported our hypothesis that NM23-H1 could be one of factors involved in the susceptibility of HNSCC cells to cisplatin. Our findings correspond closely to previous researches on melanoma, esophageal, and triple-negative breast cancers, showing that high NM23-H1 expression correlated with favorable response to chemotherapy [9-11]. The NM23-H1 expression of SAS cells did not significantly affect their growth kinetics, parallel with the conclusion that NM23-H1 silencing doesid not provide an intrinsic proliferative advantage to cancer cells but instead induces chemoresistance [28]. Hence we suggest that the effect of NM23-H1 on cisplatin cytotoxicity isnot directly related to cell proliferation, but instead presumably to the DNA damage response in our tested cell line.

Concerning the mechanism of action of cisplatin, no early apoptotic DNA fragmentation was detected after treatment, indicating that classical apoptosis may be not the major pathway responsible for cisplatin-induced death of SAS cells. Nevertheless, we cannot exclude the possibility that NM23-H1 could have a part in the caspase-independent apoptotic pathway in which DNA is damaged by single-strand nicks under certain circumstances [29]. Alternatively, we noted that most SAS cells exhibited growth arrest following cisplatin exposure, compatible with previous evidence [30].

Since the experimental data illustrated that the NM23-H1 level increased in S-phase and cisplatin had a high cytotoxicity in S-G2 phase, NM23-H1 may modulate the cell cycle in response to cisplatin-induced events [26, 31]. Without cisplatin exposure, there was no significant difference in cell cycle distribution among the SAS clones established by knockdown and overexpression of NM23-H1 [10]. Therefore, we propose that the effect of NM23-H1 on cell cycle progression could be elicited by cisplatin and involved in susceptibility of SAS cells to its damage. Most anticancer agents exert their cytotoxicity through the induction of cell cycle arrest, successively proceeding toward cell death if extensive damage occurs beyond repair. Following exposure to cisplatin, SAS cells primarily accumulated in S-phase, congruent with the fact that SAS cells can perform normal p53 functions including checkpoint activation [32]. It is well documented that NM23-H1 positively regulates p53 activities, thus NM23-H1 may participate in cisplatin-induced cell arrest [33, 34]. Knockdown of NM23-H1 delayed S-phase block until the later time point following cisplatin treatment, simultaneously with more cells in G2-M phases. It is quite probable that a portion of NM23-H1-knockdown cells may recover from the damage caused by cisplatin and then progress to G2 phase.

We discovered that the NM23-H1 level of SAS cells was upregulated upon cisplatin treatment in a time- and concentration-dependent manner, with the response of NM23-H1-overexpressing cells being the most pronounced. In response to DNA damage, accumulation of NM23-H1 in cancer cells was reported to occur concomitant with its nuclear translocation [22]. Ferguson *et al.* demonstrated that enhanced cisplatin sensitivity correlated with increased DNA cross-links in NM23-H1-overexpressing transfectants [9]. Moreover, a recent article pointed out that the 3′-5′ exonuclease activity of NM23-H1 could be involved in chromosomal instability in esophageal cancers [35]. It is most likely that intensified exonuclease activity of NM23-H1 following its overexpression may expand cisplatin-induced DNA damage and subsequently trigger more cell death [20, 36]. On cytometric analysis, we noticed that a significant increase in the S-phase fraction followed cisplatin-induced NM23-H1 accumulation in SAS cells, implying that accumulated NM23-H1 proteins may take part in activation of the S-phase checkpoint. Thus, we presume that upregulation of NM23-H1 in response to DNA damage may concurrently alter cyclin expression and result in cell cycle arrest, thereby actng as a tumor suppressor gene [37].

Certain reports have validated NM23-H1 modulation of gene expression in cell cycle regulation [38, 39]. Pharmacologic studies demonstrated that overexpression of cyclin E rendered cancer cells more susceptible to S-phase-targeted therapies in a panel of various cancer cell lines [40, 41]. In SAS cells, overexpression of NM23-H1 upregulated cyclin E and further augmented cisplatin-induced cyclin E accumulation, plausibly contributing to increased chemosensitivity. High cyclin E expression may assist the surviving cells in S-phase re-entry from chemotherapy-induced quiescence and then boost cisplatin cytotoxicity in S-phase [42]. On the other hand, knockdown of NM23-H1 downregulated cyclin E and rendered SAS cells less susceptible to cisplatin. Nonetheless, thorough investigation of the interplay between NM23-H1 and cyclin E is necessary to elucidate the connection with the response of cancer cells to cisplatin.

NM23-H1 expression has been demonstrated throughout the cell cycle with a high level in the late G1-early S and G2-M phases, in accordance with the active period of cyclin A [43]. Knockdown of NM23-H1 downregulated cyclin A in SAS cells, consistent with the observation of a low cyclin A level in the hepatoma of transgenic *NM23-M1* knockout mice. Cisplatin treatment concentration-dependently reduced cyclin A expression in SAS cells with the exception of NM23-H1-knockdown cells that maintained a relatively low cyclin A level, further inferring an association between NM23-H1 and the cyclin A checkpoint [44]. Based on clinical data, Volm *et al.* presented that reduced protein levels of both NM23 and cyclin A correlated with chemoresistance of non-small cell lung cancers [45]. Patients with high-cyclin A tumors had more favorable prognosis and a better response to chemotherapy in various cancers [46, 47]. Growing laboratory evidence also revealed that overexpression of cyclin A could have an additional effect on the cell cycle arrest and apoptosis induced by anticancer agents [48, 49]. Compatible with these results, NM23-H1-knockdown SAS cells showing a low cyclin A level exhibited less susceptibility to cisplatin compared with mock controls. Collectively, these findings may denote a potential partnership between NM23-H1 and cyclin A with regard to therapeutic efficacy of cisplatin.

In SAS cells, overexpression of NM23-H1 slightly downregulated cyclin D1 and B1 whereas knockdown of NM23-H1 upregulated them, consistent with published reports in other types of cancer cells. Several studies mentioned that elevated expression of cyclin D1 or B1 conferred chemoresistance, while decreased expression enhanced the sensitivity of cancer cells to cisplatin [50, 51]. Similarly, there was a reduction in cisplatin-induced death of NM23-H1-knockdown SAS cells that displayed an increase in the level of cyclin D1 and B1 before treatment [52-54]. In NM23-H1-knockdown cells, increased cyclin B1 protein may help some cells to overcome the barriers of cisplatin-induced S-phase arrest and keep them cycling, leading to increased cell viability. However, beyond the scope of the present study, additional experiments are needed to clarify the cross talk between NM23-H1 and cyclin D1 and B1, as well as its role in the causal link with cisplatin cytotoxicity.

In summary, our clinical data provided evidence that relatively low tumor NM23-H1 expression was associated with locoregional recurrence after postoperative cisplatin-based chemoradiation, further indicative of poor prognosis of HNSCC patients with cervical metastases. Based on the *in vitro* study of stable HNSCC SAS clones established by knockdown and overexpression of NM23-H1, we demonstrated that suppression of NM23-H1 expression attenuated cisplatin cytotoxicity, whereas elevation of NM23-H1 enhanced chemosensitivity. Following cisplatin exposure, we observed the cellular accumulation of NM23-H1 protein with a concomitant increase in the S-phase fraction of SAS cells. In addition, knockdown of NM23-H1 downregulated cyclin E and A whereas overexpression of NM23H1 upregulated them in SAS cells. These findings suggest that NM23-H1 may participate in cellular susceptibility to cisplatin through modulation of S-phase regulators. However, since our results may reflect a cell line-specific phenomenon, we will confirm their validity by testing other human HNSCC cell lines. Future research is necessary to scrutinize the link between NM23-H1 and cell cycle regulators in the response of HNSCC cells to chemoradiation. As a clinical application, increasing tumor NM23-H1 expression may be a potential strategy to improve the efficacy of traditional cisplatin-based therapy for HNSCC patients with metastases.

## MATERIALS AND METHODS

### Patients and tissue specimens

The tissue specimens were obtained between 1984 and 1998 from 46 patients with HNSCC and resectable cervical metastases treated by postoperative cisplatin-based chemoradiation. The patient ages ranged from 24-70 years with a median of 45 years. All patients provided written informed consent and this study was approved by the Institutional Review Broad of Taipei Veterans General Hospital. The preoperative work-up consisted of physical examination, intraoral biopsy, computed tomography (CT) scan of the head and neck, sonography of the abdomen, chest radiography and whole-body radioisotopic bone scan. We followed the same treatment plan for all patients, who underwent surgical removal of primary cancers and cervical metastases. Locoregional irradiation and systemic cisplatin-based chemotherapy were postoperatively administered. The cancer stages were categorized based on the TNM system of the American Joint Committee on Cancer [55]. Tumor specimens were obtained during the therapeutic surgeries and normal counterpart specimens were excised from the neighboring grossly disease-free mucosa of the surgical margins. Both tumorous and nontumorous tissues were confirmed by pathologists (Li WY). Postoperative cisplatin-based chemoradiotherapy was administered for all patients because of close surgical margins, multiple metastatic lymph nodes, extracapsular spread, or perineural invasion. Tumor recurrence was defined as when clinical examinations showed evidence of recurrence during the regular follow-up. The median follow-up period was 30 months with a range of 3-218 months. Up to the time of final statistical analysis, 18 of 46 patients were alive and free of HNSCC. The overall cumulative 1-, 3- and 5-year survival rates were 54%, 48%, and 45%, respectively.

### Immunohistochemistry and scoring

Expression of NM23-H1 in the pathologic sections was detected by an immunoperoxidase method as previously described [6, 17]. Paraffin blocks were sectioned at the thickness of 4 μm. The wax was melted at 65°C overnight. The sections were deparaffinized in xylene, which was subsequently removed with absolute ethanol. The slides were incubated with specific mouse monoclonal antibodies against NM23-H1 (Santa Cruz Biotechnology, Dallas, TX) followed by biotin-conjugated goat anti-mouse immunoglobulin and horseradish peroxidase (HRP)-conjugated streptavidin (Dako, Glostrup, Denmark). Aminoethylcarbazole was used as a chromogenic substrate and red precipitate was identified as positive staining. The specimens were counterstained with hematoxylin and mounted with glycerol gelatin. In each experiment, a section of human breast cancer known to overexpress NM23-H1 served as a positive control (Dako) and a section with an immunoglobulin class-matched nonimmune antibody substituted for the primary antibody by an equivalent staining protocol used as a negative control. Each batch of immunohistochemistry experiments contained both positive and negative controls to ensure staining quality.

Slide evaluation has been described previously [6, 17]. Briefly, the histologically non-tumorous mucosa served as the internal negative control for each case. Under a low-power field, ten different regions of each slide containing tumor cells were randomly evaluated independently by two investigators (Li WY and Wang YF) unaware of the clinical data and at least 100 tumor cells were examined per field. Two scoring systems, the staining intensity and percentage of stained cells, were included in our study. Staining intensity was scored on a semiquantitative four-point scale as follows: 0, equivalent to the negative control; 1, weak staining slightly darker than the negative control; 2, moderate staining of an intensity between scores 1 and 3; and 3, intense staining equivalent to or darker than the positive control. We used photomicrographs representing the four scores (0-3) as standards while interpreting the slides. When >5% of cancer cells showed nuclear staining and >25% had cytoplasmic staining intensity scores >2, the sample was considered NM23-H1 positive. In the discrepant cases, a final opinion was made based on consensus of the two investigators.

### HNSCC cell line and culture conditions

The human HNSCC cell line, SAS, was obtained from the Japanese Collection of Research Bioresources (JCRB, Tokyo, Japan). HNSCC cells were cultivated in 5% CO_2_ at 37^o^C in Dulbecco modified Eagles' medium (DMEM) supplemented with 10% fetal bovine serum and antibiotics (100 U/mL penicillin, 100 μg/mL streptomycin). The culture media were obtained from Gibco Laboratories (Life Technologies, Carlsbad, CA).

### Plasmids and transfection

To generate the nm23-H1 siRNA expression vectors, we followed the algorithm for siRNA design based on the pSuper RNAi system protocol (Oligoengine, Seattle, WA) [17]. Forward and reverse sequences for anti-nm23-H1 siRNA construct were as follows: 5′ GAT CCC CTG CAA GCT TCC GAA GAT CTT TCA AGA GAA GAT CTT CGG AAG CTT GCA TTT TTA3'; 5′AGC TTA AAA ATG CAA GCT TCC GAA GAT CTT CTC TTGAAA GAT CTT CGG AAG CTT GCA GGG3'. These two 60-nucleotide-containing oligos were chemically synthesized and annealed to form a duplex. The annealed oligos were then ligated into the BglII-HindIII restriction sites within the pSuper vector, yielding pSuper.nm23-H1. To establish NM23-H1 knockdown and the corresponding mock HNSCC clones, pSuper.nm23-H1 and empty pSuper plasmids were transfected into SAS cells using Lipofectamine2000 (Invitrogen, Carlsbad, CA) according to the manufacturer's instructions. Stable clones were selected under Geneticin (G418, 700 g/mL; Life Technologies) treatment for 14 days to allow colony formation. Colonies resistant to G418 were isolated with cloning cylinders. Individual NM23-H1 knockdown (SAS_shRNAnm23_) and mock (SAS_shRNA_) clones were amplified for further analyses.

To construct the NM23-H1 expression vector, we subcloned the full-length cDNA of nm23-H1 tagged the hemagglutinin (HA) into the BamH1 and EcoRI sites of the pcDNA3 (Invitrogen) plasmid. SAS cells were transfected with pcDNA3-HA-nm23-H1 or pcDNA3 vector alone [17]. To select the stable transfectants, these cells were treated with G418 for 14 days to allow colony formation. Stable SAS clones overexpressing the exogenous HA-NM23-H1 protein (overexpression clone; SAS_nm23_) and those containing pcDNA3 vectors alone (mock overexpression clone; SAS_control_) were confirmed by Western blot analysis.

### Protein extraction and Western blot analysis

Proteins were extracted from the subconfluent cultures of SAS clones. Cultured cells were washed twice in phosphate-buffered saline (PBS) and lysed in a buffer containing 50 mM Tris (pH 6.8), 150 mM NaCl, 1 mM disodium EDTA, 5% β-mercaptoethanol, 1 mM phenylmethyl-sulfonylfluoride, 0.01% bromophenol blue, 10% glycerol and 1% sodium dodecyl sulfate supplemented with leupeptin (10 μg/mL), aprotinin (10 μg/mL) and protease inhibitors (10 μg/mL). The protein content was determined by the Bradford method (Bio-Rad Laboratories, Hercules, CA). Western blot analysis was performed as described previously [56]. The membrane was probed with the specific mouse monoclonal antibodies against NM23-H1 (Santa Cruz Biotechnology; 1: 50), -actin (Santa Cruz Biotechnology; 1: 5000), cyclin D1 (MBL International, Woburn, MA; 1: 1000), cyclin E (MBL International; 1: 1000), cyclin A (Cell Signaling Technology, Danvers, MA; 1: 1000) or cyclin B1 (Cell Signaling Technology; 1: 1000), and then HRP-conjugated goat anti-mouse IgG (Jackson ImmunoResearch Inc., West Grove, PA; 1:5000). Immunoreactive bands were visualized using the enhanced chemiluminescence detection reagents (Immobilon, Millipore Co., Billerica, MA).

### Cell growth and viability by trypan blue exclusion assay

SAS clone cells were plated in 10-cm dishes at a density of 2.0 × 10^5^ cells/dish. Once daily, cells were washed twice with PBS and trypsinized using 1 mL trypsin-ethylenediamine tetraacetic acid (0.05% trypsin, 0.53 mM ethylenediamine tetraacetic acid•4Na (Gibco/Invitrogen). Suspended cells were resuspended in fresh culture medium and stained with a 0.4% solution of trypan blue (Gibco/Invitrogen). Cell growth was determined with hemocytometer-based cell quantification using a trypan blue dye exclusion assay. The doubling time was calculated from the cell growth curve plotted over 4 days.

To assess the chemosensitivity to cisplatin, the SAS clones were plated in 10-cm dishes at a density of 5.0 × 10^5^ cells/dish and incubated with various concentrations of cisplatin (0, 1, 3, 10, and 30 μM). After a 48-hour treatment, cells were subjected to the aforementioned trypan blue dye exclusion assay.

### Flow cytometry for cell cycle analysis and NM23-H1 staining

Subconfluent cultures of the SAS clones were trypsinized, collected, and washed twice with PBS. Cells were resuspended in PBS, fixed in 70% ethanol, and stored at 4°C. On the day of analysis, the prepared samples were analyzed according to previously reported methods [57, 58]. For each measurement, a minimum of 15,000 cells were analyzed. All data were analyzed using offline software (FlowJo version 7.6.5, Tree Star Inc., Ashland, OR).

To detect NM23-H1-positive cells, 5 × 10^5^ cells were fixed, permeabilized, and incubated with a mouse anti-NM23-H1 antibody or isotype control (BD Biosciences, Franklin Lakes, NJ) at 4°C for 30 minutes. Cells were washed, resuspended, and incubated with an anti-mouse immunoglobulin G fluorescein isothiocyanate (FITC)-conjugated antibody (Dako) at 4°C for 20 minutes. Cells were washed and resuspended in staining solutions A and B for 30 minutes before flow cytometric analysis.

### Statistical analysis

To examine the relationships between NM23-H1 expression and each clinicopathologic parameter, statistical analyses were performed with Chi-square (χ2) tests with Yates correction or Fisher's exact test. For prognostic analyses, the survival curves were plotted using the Kaplan−Meier method. The statistical difference of survival between the patient groups was compared using log-rank test. The joint effects of clinicopathologic factors were further tested in the multivariate analysis using a Cox proportional hazards model.

For *in vitro* studies, data are shown as the mean ± standard error of at least three independent experiments except where indicated. Differences between groups at each time-point were identified by one-way analysis of variance (ANOVA) or Wilcoxon-signed rank test. Statistical comparison between two independent variables was determined by two-way ANOVA followed by Dunnet's test. Statistical analysis was performed using Statistical Package of Social Sciences (SPSS) software (SPSS Inc., Chicago, IL). Probability *P*-values < 0.05 were considered statistically significant.
